# Rationalisation and visualisation of non-bonded interactions

**DOI:** 10.1186/1758-2946-5-S1-P48

**Published:** 2013-03-22

**Authors:** Stephen Maginn, Paul Labute, Alain Ajamian, Chris Williams

**Affiliations:** 1 Chemical Computing Group, St Johns Innovation Centre, Cambridge CB4 0WS, UK; 2 Chemical Computing Group, 1010 Sherbrooke Street West, Montréal, Québec H3A 2R7, Canada

## 

Models of non-bonded interactions are crucial in structure-based drug design. "Standard" hydrogen bonds are well modelled through traditional molecular mechanics forcefields with their treatments of electrostatics, and functional forms, often based on abundant crystal structure data, to describe their geometries. But "non-standard" interactions - for example, hydrogen bonds with carbon as the donor, or so-called halogen interactions - are not well handled, or not handled at all.

Here, we describe the use of extended Hückel (E-Hückel) theory, a very fast, low-level MO theory, to evaluate the geometries and energies of such non-standard interactions. These include successful modelling of halogen interactions for Cl, Br and I, which each display the so-called "sigma hole" in QM calculations, and likewise interactions between sulphur and oxygen. The E-Hückel modelling has been implemented in the 2011.10 version of the MOE (Molecular Operating Environment) software system.

**Figure 1:**
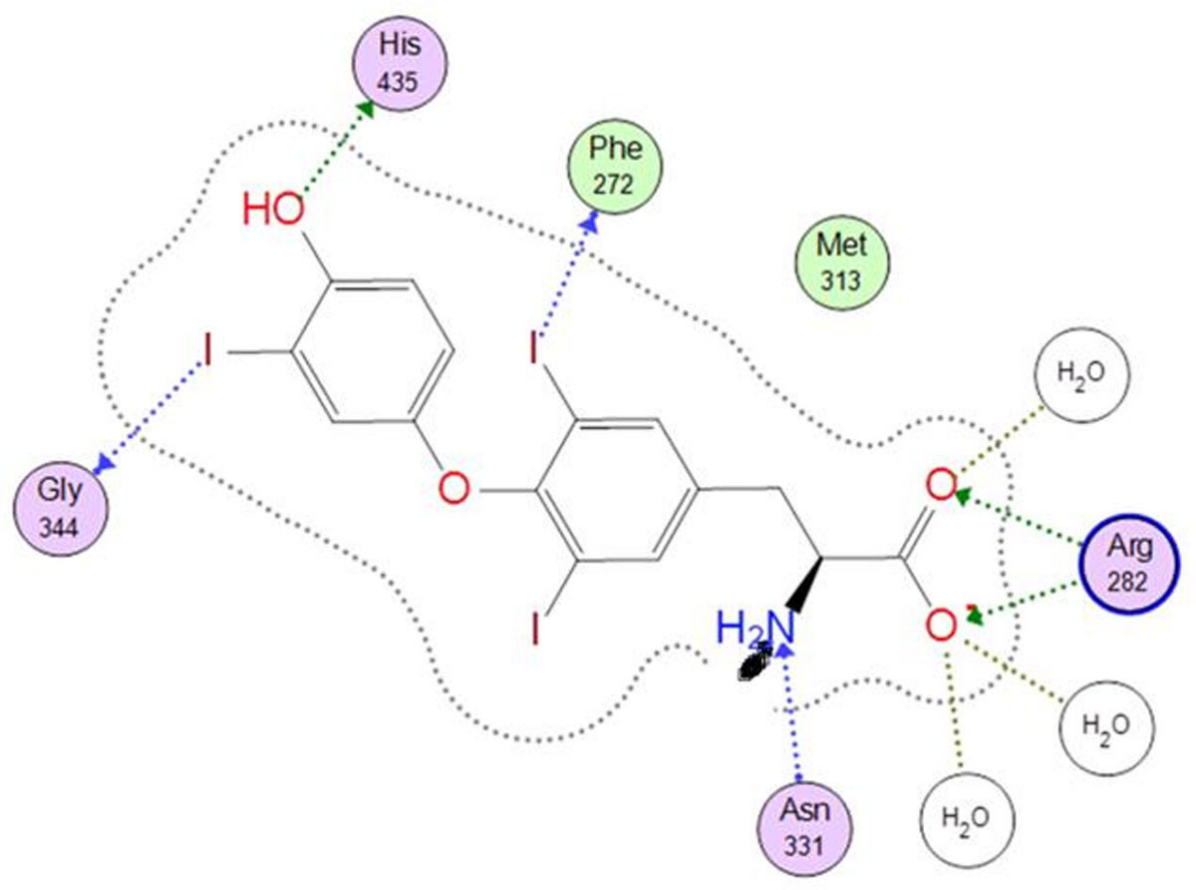
**Triiodothyronine (T3) in its receptor binding site**. (PDB 3GWS).

A pharmacophore feature type has been created for halogen interactions; an example of its use in finding scaffold replacements for thyronines (thyroid hormones), which employ halogen interactions involving iodine (Figure 1) [1], will be presented.
